# Development of fluconazole resistance in a series of *Candida parapsilosis* isolates from a persistent candidemia patient with prolonged antifungal therapy

**DOI:** 10.1186/s12879-015-1086-6

**Published:** 2015-08-18

**Authors:** Li Zhang, Meng Xiao, Matthew R. Watts, He Wang, Xin Fan, Fanrong Kong, Ying-Chun Xu

**Affiliations:** Department of Clinical Laboratory, Peking Union Medical College Hospital, Chinese Academy of Medical Sciences, Beijing, 100730 China; Graduate School, Peking Union Medical College, Chinese Academy of Medical Sciences, Beijing, 100730 China; Centre for Infectious Diseases and Microbiology Laboratory Services, ICPMR – Pathology West, University of Sydney, Westmead Hospital, Darcy Road, Westmead, Sydney, NSW 2145 Australia

**Keywords:** *Candida parapsilosis*, Fluconazole resistance, Persistent candidemia, Antifungal treatment, *MDR1*, *MRR1*

## Abstract

**Background:**

*Candida parapsilosis* was the most common species causing candidemia in the 2010 China Hospital Invasive Fungal Surveillance Net (CHIF-NET) database. Compared to *Candida albicans*, the description of azole resistance and mechanisms in *C. parapsilosis* is very limited. We report a patient with *C. parapsilosis* candidemia over several months, due to a probable intravascular source, who developed fluconazole resistance after prolonged treatment.

**Case presentation:**

An 82 year-old male had a hospital admission of approximately 1.5 years duration. He was initially admitted with acute pancreatitis. Prior to succumbing to the illness, he developed candidemia and treated with three antifungal drugs for nearly 5 months, at suboptimal doses and without source control. Following treatment, 6 blood cultures were still positive for *C. parapsilosis*. The last 2 strains were resistant to fluconazole (MICs 32 μg/mL) and intermediate to voriconazole (MICs 0.5 μg/mL). Microsatellite multilocus analysis indicated that the 6 isolates from the patient belonged to a single genotype. The first 4 isolates were susceptible to fluconazole (MICs 2 μg/mL) and voriconazole (MICs 0.015-0.03 μg/mL), which were slightly higher than susceptible control strains from other patients. Overexpression of *MDR1* genes were detected in the two resistant isolates, and this was associated with a homozygous mutation in *MRR1* genes (T2957C /T2957C), with the amino acid exchange L986P.

**Conclusions:**

This case corroborates that the resistant *C. parapsilosis* isolates can emerge in the setting of complicated infections and the extensive use of antifungal agents, emphasizing the need for standardizing and improving the antifungal treatment as well as source control in the treatment of infection diseases.

## Background

*Candida parapsilosis* is a significant clinical pathogen that can grow in total parenteral nutrition, form biofilms on catheters and other implanted devices, persist in the hospital environment and be nosocomially transmitted by hand carriage [1–4]. In China, *C. parapsilosis* was the most common species causing candidemia in the 2010 China Hospital Invasive Fungal Surveillance Net (CHIF-NET) study [5].

Azoles are the most commonly used drugs for the treatment of *Candida* infections. Besides species that show intrinsic resistance, such as *Candida krusei*, the acquisition of azole resistance, particularly after prolonged exposure and prophylactic overuse, is well described in *Candida albicans*, *Candida tropicalis*, *Candida glabrata* [6–9]. However, the descriptions of azole resistance in *C. parapsilosis* are very limited [10, 11].

Constitutive overexpression of 2 types of multidrug efflux pumps, encoded by *CDR1* or *MDR1* genes is a major cause of resistance to azoles [12–14]. Morschhäuser et al. found that gain of function mutations in *MRR1* genes cause constitutive *MDR1* overexpression in fluconazole-resistant *C. albicans* and *C. dubliniensis* [15–17]. Similarly, the mutations in *TAC1*, a transcription factor regulating *CDR* genes, are responsible for the constitutive high-level expression of *CDR* genes [18, 19]. Another common mechanism is the *ERG11* gene overexpression or acquisition of mutations, resulting in target enzyme up-regulation or reduced affinity to bind azoles [12–14]. The mutations in *UPC2* are a frequent cause of *ERG* upregulation [20].

We encountered a case of induced fluconazole resistance in *C. parapsilosis* from a patient with persistent candidemia due to a probable intravascular source in the Peking Union Medical College Hospital (PUMCH). Here we describe the case and explore the possible resistance mechanism.

## Case presentation

**Fig. 1:**
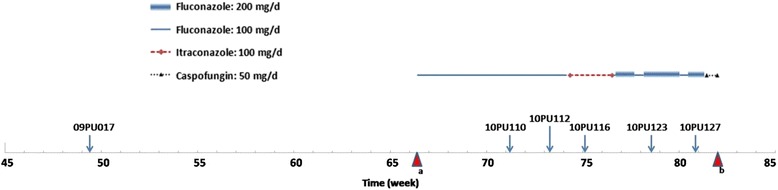
The antifungal treatment course and isolated *C. parapsilosis* strains information of the patient. ^*a*^Sputum smear showed a large amount of yeast and antifungal treatment was commenced. ^*b*^The patient died of systemic invasive fungal infection and chronic renal failure

An 82 year-old male was admitted to the PUMCH in December, 2008 with severe, acute pancreatitis. He was managed with mechanical ventilation and received parenteral nutrition, diuretics and anti-microbial therapy. His renal function deteriorated in the 11^th^ week following hospitalization, he began haemodialysis, 3 times per week, through a left internal jugular venous catheter. In the 49^th^ week, he developed a fever of 39 °C, and blood culture were collected. He was treated with empirical meropenem and his fever resolved after 3 days. His blood culture were positive for *C. parapsilosis* after 7 days, however, due to his clinical improvement he was not given any antifungal therapy (Fig. 1). In the 52^nd^ week, the left internal jugular venous catheter was removed, and an arteriovenous fistula was created in the forearm using a polytetrafluoroethylene (PTFE) graft to allow hemodialysis. In the 66^th^ week, he became febrile and a sputum smear showed a large amount of yeast, with culture positive for *C. glabrata*. CT scan of the chest showed nodules in the right upper lobe and bilateral pleural effusion. He was treated for a pulmonary fungal infection, with fluconazole (100 mg/day, renal dose adjusted) (Fig. 1). While still on the antifungal therapy, *C. parapsilosis* susceptible to fluconazole was isolated from blood culture in the 71^st^ week following admission. In the subsequent 9 weeks the patient had intermittent fevers. There were 4 other blood cultures positive for *C. parapsilosis*, consistent with an intravascular source of infection (Fig. 1). The final two isolates (PU123 and PU127) were resistant to fluconazole and had intermediate susceptibility to voriconazole.

Antifungal treatment received by the patient and isolated strains are summarized in Fig. 1. Due to fluconazole treatment failure, it was changed to intraconazole (100 mg/day) in the 74^th^ week. This was ceased in the 76^th^ week and fluconazole was recommenced at a higher dose (200 mg/day). As a possible source of infection, the synthetic arteriovenous fistula was removed in the 78^th^ week. However, the candidemia persisted and in the 80^th^ week caspofungin (50 mg/day) was commenced.

In the 81^st^ week, the patient became comatosed (Glasogow Coma Scale 5) with hypertonia, a positive Babinski sign, and neck rigidity, indicative of central nervous system infection or metabolic encephalopathy. No further investigations were performed, as treatment was withdrawn following discussion with the patient’s family. The patient died in the 82^nd^ week of admission.

### Comparison with control isolates

The use of the isolates in the present study was approved by the Human Research Ethics Committee of Peking Union Medical College Hospital (No. S-263). We have obtained the writing consent from the control patients to publish the case report.

There were 6 blood culture isolates from the patient described in the case study (patient 1). In addition, 6 isolates of *C. parapsilosis* obtained from the other patients (patients 2–7) in the PUMCH during the hospitalization of patient 1 were studied for control purpose. Identification of *C. parapsilosis* was confirmed by DNA sequencing of the fungal internal transcribed spacer (ITS) region and the D1/D2 domain of the 28S rRNA gene, using a published protocol [5].

Sensititre YeastOne YO10 broth microdilution susceptibility panels (TREK Diagnostic Systems, Westlake, Ohio) were used to test the susceptibility of *C. parapsilosis* to antifungal agents, according to a published protocol [21]. For *C. parapsilosis,* the interpretation of fluconazole, voriconazole and three echinocandins susceptibilities was done in accordance with CLSI M27-S4 [22]. Table 1 summarizes the *in vitro* susceptibility results of the 12 *C. parapsilosis* isolates. There was no significant difference in the drugs tested except for fluconazole and voriconazole. The isolates from patients 2–7 were susceptible to fluconazole (MICs 0.25-0.5 μg/mL) and voriconazole (MICs ≤0.008 μg/mL) (Table 1). For patient 1, isolates PU017, PU110, PU112 and PU116 were susceptible to fluconazole (MICs 2 μg/mL) and voriconazole (MICs 0.015-0.03 μg/mL), but with higher MICs than the other susceptible strains. Isolates PU123 and PU127 were resistant to fluconazole (MICs 32 μg/mL) and had intermediate susceptibility to voriconazole (MICs 0.5 μg/mL) (Table 1).

**Table 1:** *C. parapsilosis* strains information, antifungal susceptibilities, microsatellite typing results and the *MRR1* gene mutations

Strain no.^a^	Patient	Ward^b^	Susceptibility results by Sensititre YeastOne^c^ (μg/mL)									Multilocus genotype^d^				Genotype^e^	*MRR1*
			FLC	VRC	ITC	POS	ANF	MCF	CAS	5FC	AMB	B5	CP1	CP4	CP6		
PU017	1	EGW	2	0.03	0.12	0.06	0.5	0.5	0.25	0.06	0.5	109/109	242/242	307/307	291/291	A	T2957C/WT
PU110	1	EGW	2	0.03	0.12	0.06	0.5	0.5	0.25	0.06	0.5	109/109	242/242	307/307	291/291	A	T2957C/WT
PU112	1	EGW	2	0.03	0.06	0.06	0.25	0.25	0.25	0.06	0.5	109/109	242/242	307/307	291/291	A	T2957C/WT
PU116	1	EGW	2	0.03	0.06	0.06	0.5	0.5	0.25	0.06	0.5	109/109	242/242	307/307	291/291	A	T2957C/WT
PU123	1	EGW	32	0.5	0.12	0.06	0.5	0.5	0.5	0.06	0.5	109/109	242/242	307/307	291/291	A	T2957C/T2957C
PU127	1	EGW	32	0.5	0.12	0.06	0.5	0.5	0.5	0.06	0.5	109/109	242/242	307/307	291/291	A	T2957C/T2957C
PU004	2	Outpatient	0.5	≤0.008	0.06	0.03	0.5	0.5	0.25	0.06	0.5	107/107	242/242	307/307	267/267	B	WT/WT
PU026	3	ICU	0.25	≤0.008	0.03	0.03	0.5	0.5	0.25	0.06	0.5	143/145	242/242	304/304	282/282	C	WT/WT
PU090	4	Medical ward	0.5	≤0.008	0.03	0.03	0.5	0.5	0.25	≤0.06	0.5	113/129	224/245	358/358	267/270	D	WT/WT
PU106	5	Surgical ward	0.5	≤0.008	0.06	0.03	0.5	0.25	0.12	0.06	0.5	109/109	242/242	307/307	291/291	A^f^	WT/WT
PU108	6	EGW	0.5	≤0.008	0.06	0.03	0.5	0.5	0.25	0.12	0.5	115/129	242/248	364/385	267/267	E	WT/WT
PU131	7	Medical ward	0.5	≤0.008	0.03	0.015	0.5	0.5	0.25	0.12	0.5	109/109	242/242	307/307	288/288	F	WT/WT
ATCC 22019	-	-	1	0.015	0.12	0.03	0.5	0.5	0.25	0.12	0.5	129/129	245/251	304/304	291/291	G	WT/WT

^a^All strains were isolated from blood cultures

^b^ *EGW* Emergency general ward

^c^ *FLC* Fluconazole, *VRC* Voriconazole, *ITC* Itraconazole, *POS* Posaconazole, *ANF* Anidulafungin, *MCF*, Micafungin, *CAS* Caspofungin, *5FC* 5-Flucytosine, *AMB* Amphotericin B. Clinical breakpoints for susceptible, intermediate, and resistant for *C. parapsilosis*, respectively, were those of the CLSI M27-S4 for fluconazole (≤2/4/ ≥8 μg/mL); and for voriconazole (≤0.125/0.25/≥1 μg/mL); and for anidulafungin, caspofungin, and micafungin (≤2/4/≥8 μg/mL)

^d^The numbers indicate the fragment size in base pairs of the different alleles obtained with the listed marker

^e^The genotype was designated according to the different microsatellite typing results

^f^The PU106 isolate yielded identical genotype with the isolates from patient 1

All the strains were genotyped using the highly polymorphic microsatellite markers, B5, CP1, CP4 and CP6 [23]. Amplification reactions were performed as previously reported [23]. The microsatellite multilocus genotypes allowed the differentiation of the 12 strains from 7 patients into 6 different genotypes. The 6 isolates from patient 1 involved a single genotype (genotype A). The strains isolated from control patients 2–4 and 6, 7 were assigned to genotypes B to G based on the observed differences. Isolate PU106 from the control patient 5 showed the same pattern on microsatellite sequence analysis as the isolates from patient 1 and designated genotype A (Table 1).

Primers used for PCR amplification of *MRR1, TAC1, UPC2,* and *ERG11* genes were listed in Table 2 (11). After alignment of the *MRR1* sequences from the 12 isolates, a single nucleotide mutation (T2957C/WT) was detected in PU017, PU110, PU112 and PU116 compared with the *MRR1* sequence of *C. parapsilosis* ATCC 22019 and the control strain with the same pattern on microsatellite typing, PU106 genotype A (Table 1). This mutation results in the replacement of a leucine amino acid residue with a proline (L986P). In the PU123 and PU127 isolates from patient 1, mutations were found in both alleles (T2957C/T2957C). After alignment of *TAC1, UPC2*, and *ERG11* gene sequences from the 12 isolates, no mutation was found.

**Table 2:** Sequences of primers used in genes sequencing

Primer name	Primer sequence	Reference
MRR1-F	5'-CCCTTTCTTCCGCAGATTTC-3'	11
MRR1-R	5'-CGTTGTAAAGATGGCGTGGT-3'	
TAC1-F	5'-AAGAGACCTACAGATAGTGC-3'	
TAC1-R	5'-CTTGAGATGCTGAGACATAT-3'	
UPC2-F	5'-TTCGTGATAGTTTTGGTGGTAG-3'	11
UPC2-R	5'-TTTCCTCCACCCCTATTGTAG-3'	
ERG11-F	5'-ATGGCATTAGTTGATTTAGCCCT-3'	
ERG11-R	5'-TCAGATTACACATGTATCTCTTT-3'	

**Fig. 2:**
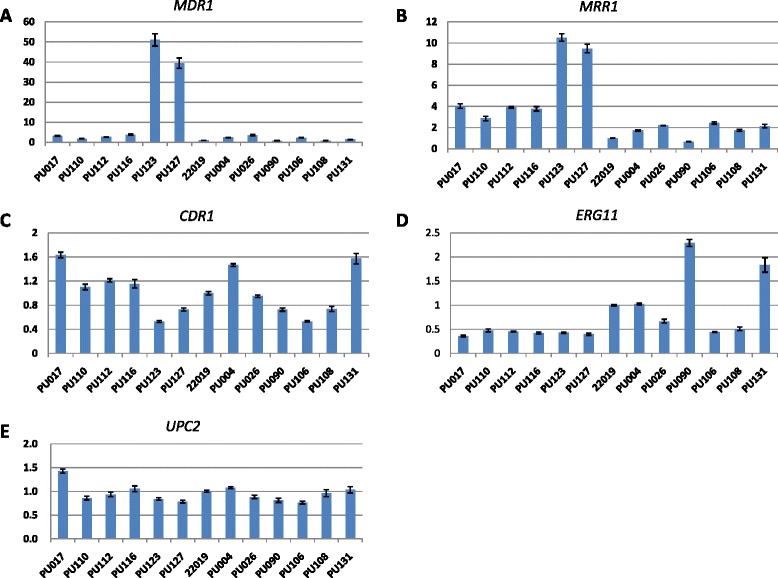
Quantitative real-time RT-PCR analysis of *MDR1* (**a**)*, MRR1* (**b**)*, CDR1* (**c**)*, ERG11* (**d**) and *UPC2* (**e**) genes expression levels in the 6 isolates from patient 1 and 6 susceptible isolates from other patients. Each sample was processed in triplicate. Error bars show the standard deviations

Overnight *C. parapsilosis* cultures were diluted to an optical density at 600 nm (OD_600_) of 0.2 in YPD medium and then incubated at 35 °C with shaking at 150 rpm for additional 6 h to mid-log phase. Total RNA was extracted from isolates grown in YPD medium using the Yeast RNAiso Reagent Kit (TaKaRa, Tokyo, Japan) and reverse transcribed to cDNA using the PrimeScript RT Reagent Kit (TaKaRa, Tokyo, Japan) according to the instructions of the manufacturer. The quantitative real-time RT-PCRs were performed in triplicate using the SsoFast EvaGreen supermix (Bio-Rad, Hercules, CA, USA) on a BioRad CFX96 system. ATCC 22019 were used as the control isolate. The primers used in this study were listed in Table 3 [11, 24, 25]. The *ACT1* gene was used as the endogenous control. The change in fold expression was obtained by calculating 2^-ΔΔ*CT*^, and a change of 2.5 times was considered to be overexpressed [26]. The 2 resistant isolates from patient 1 had higher expression levels of *MRR1* than the four susceptible isolates from patient 1 (PU123 10.5-fold and PU127 9.5-fold; Fig. 2). The 4 susceptible isolates showed higher expression levels (>2.5 fold) than the controls. The *MDR1* expression was further increased in the resistant isolates (PU123 51.0-fold and PU127 39.4-fold). In contrast, the *CDR1*, *UPC2*, *ERG11* genes expression levels in the 2 azole-resistant isolates were not significantly different from the 4 susceptible isolates from patient 1 and the control isolates.

**Table 3:** Sequences of primers used in quantitative real-time RT-PCR

Primer name	Primer sequence	Reference
MRR1-F	5'-ACAATGGTCTGAGCAATGAA-3'	11
MRR1-R	5'-GGCAATACTGGTGATGGAA-3'	
MDR1-F	5'-TTCGTGATAGTTTTGGTGGTAG-3'	11
MDR1-R	5'-TGAACCTGGAGTGAATCTTGT-3'	
CDR1-F	5'-GCGTTTGACCATCGGAGTT-3'	24
CDR1-R	5'-TACCGCTGTTTGCGAATCT-3'	
UPC2-F	5'-ATTGGAGTGTGGGTATCTTCAT-3'	11
UPC2-R	5'-CCTTCGCCTTCTTCAGTTC-3'	
ERG11-F	5'-GGTTTACTTGTGTTTGCTCCT-3'	11
ERG11-R	5'-GTCCATAAGATACGGCTGAAC-3'	
ACT1-F	5'-ATGATAGAGTTGAAAGTAGTTTGGTCAATA-3'	25
ACT1-R	5'-ACTACTGCTGAAAGAGAAATTGTTAGAGAC-3'

In order to confirm that only *MRR1* and *MDR1* genes were overexpressed, we subcultured isolates in the presence of sub-inhibitory concentrations of fluconazole, and repeated the gene expression studies. Two susceptible isolates (PU017, PU116), the 2 resistant isolates (PU123 and PU127) from patient 1, 3 control isolates (PU090, PU106 and PU131) were subcultured. All the tested isolates and the control strain ATCC 22019 were firstly incubated at 37 °C for 4 h in RPMI 1640 (Sigma, USA). Fluconazole was then added at a concentration of 1/2 MIC and the isolates were incubated for an additional 4 h. RNA extraction and the quantitative real-time RT-PCRs were performed as the previous experiment in the present study.

The 2 resistant isolates showed higher expression levels of *MRR1* than the other isolates (PU123 3.90-fold and PU127 3.99-fold; Table 4). The *MDR1* expression was also obviously increased in the resistant isolates (PU123 9.70-fold and PU127 9.01-fold). Similar to the previous experiment, the *CDR1*, *UPC2*, *ERG11* genes expression levels in the 2 azole-resistant isolates were not higher than other susceptible isolates.

**Table 4:** Gene expression in the eight *C. parapsilosis* isolates cultured in the presence of fluconazole with concentrations of 1/2 MIC

Sample	*MDR1* ^*b*^		*MRR1* ^*b*^		*CDR1* ^*b*^		*ERG11* ^*b*^		*UPC2* ^*b*^	
	Expression	Expression SEM	Expression	Expression SEM	Expression	Expression SEM	Expression	Expression SEM	Expression	Expression SEM
ATCC22019^a^	1.00	0.04	1.00	0.03	1.00	0.02	1.00	0.04	1.00	0.03
PU017	2.38	0.09	1.74	0.04	0.81	0.03	0.87	0.02	1.04	0.03
PU116	2.33	0.13	1.55	0.10	1.03	0.06	1.08	0.06	1.16	0.06
PU123	9.70	0.63	3.90	0.20	0.21	0.01	0.26	0.15	0.31	0.02
PU127	9.01	0.28	3.99	0.14	0.28	0.01	0.35	0.01	0.37	0.02
PU090	2.16	0.10	0.74	0.03	0.77	0.03	0.55	0.03	0.53	0.02
PU106	1.45	0.07	1.55	0.05	1.55	0.04	1.13	0.05	1.86	0.07
PU131	1.17	0.04	1.31	0.05	0.95	0.02	0.52	0.01	0.52	0.02

^a^ATCC22019 isolates was used as control isolate, and was also cultured in the presence of 1/2 MIC of fluconazole

^b^All the samples for each gene were tested in triplicate. The left column shows the mean value of expression, and the right column shows the Standard Error of Mean (SEM)

## Discussion

Compared to other *Candida* species, *C. parapsilosis* tends to be associated with higher MICs to echinocandins [27]. Therefore, the development of azole resistant *C. parapsilosis* has significant clinical implications, as multiazole- and multiechinocandin-resistant isolates would limit available treatment options [10]. In addition, there could be nosocomial transmission of resistant *C. parapsilosis* between vulnerable patient groups [1–4].

Microsatellite genotyping was consistent with fluconazole resistant developing in previously susceptible strains of *C. parapsilosis*. The 2 resistant patient-isolates (PU123 and PU127) overexpressed *MRR1* and *MDR1*. The *MRR1* overexpression was highly associated with mutation (T2957C), leading to the amino acid exchange, L986P. This indicates that the *C. parapsilosis* resistance to fluconazole was conferred by the increased expression of the *MRR1* transcription factor, which resulted in a concomitant overexpression of *MDR1*.

The previous studies have demonstrated that a gain-of-function mutation in one *MRR1* allele results only in slightly decreased susceptibilities to the azoles and the loss of heterozygosity further increases drug resistance [15, 16]. A similar observation was made in the present study, where the patient isolates that were heterozygous *MRR1* (T2957C/WT) mutants showed slightly higher MICs than wild type strains (WT/WT). The last 2 isolates became homozygous (T2957C/T2957C) mutants and showed much higher MICs and overexpression of *MDR1*. While this mutation was the probable cause of the phenotypic resistance in this case, a mutagenesis study that demonstrated the development of resistance and increased gene expression in a previously sensitive organism would provide additional evidence.

After repeating the experiment in which the strains were cultured in media containing fluconazole with concentrations of 1/2 MIC for each isolate, we confirmed that only *MDR1* and *MRR1* were overexpressed in 2 resistant isolates. However, *MDR1* expression did not increase as much as in the initial experiment. This may be related to different medium as well as all the isolates, including the control isolate ATCC22019, being exposed to fluconazole. The *ERG11* genes in resistant isolates were not overexpressed, even following to fluconazole. Similarly, Grossman et al. has sequenced the *ERG11* and *MRR1* genes in 30 fluconazole resistant *C. parapsilosis* isolates and 37 susceptible dose-dependent isolates, and found no isolate with both the *MRR1* and *ERG11* gene mutation [28]. In most cases, phenotypes of resistance developed due to a combination of mechanisms, so the whole genome sequencing will be included in subsequent investigation.

This case corroborates that the resistant *C. parapsilosis* isolates can emerge in the setting of complicated infections and the extensive use of antifungal agents. Patient 1 received fluconazole treatment for more than 3 months. However, treatment was not instituted for the first candidemia and when fluconazole treatment was commenced it was initially at a lower dose because of chronic renal failure, and on the basis of sputum microscopy. In the previous studies, suboptimal fluconazole dosing has leaded to the development of resistance in *Candida* species [6, 29]. In addition, itraconazole was used for 2 weeks, when it is not recommended for invasive candidiasis [30]. This highlights the need for standardization of antifungal treatment, in terms of drug selection, dose and duration [31].

In this case, probable sources of the persistent candidemia were the intravenous hemodialysis catheter and the synthetic vascular graft. Vascular catheters have been regarded as the source in more than 50 % of cases of *C. parapsilosis* candidemia and prompt removal of the catheter is recommended [32].

## Conclusions

This report described a case where fluconazole resistant *C. parapsilosis* emerged during prolonged antifungal treatment, with associated *MDR1* overexpression, which was related to a *MRR1* mutation (T2957C). Resistant *C. parapsilosis* has the potential to complicate the management of candidemia in vulnerable patient groups. This case illustrates the need for effective antifungal treatment, source control in the treatment of infection diseases.

### Consent

Written informed consent was obtained from the patient for publication of this Case report. A copy of the written consent is available for review by the Editor of this journal.
